# *Mycobacterium tuberculosis* Lipoprotein LprG Binds Lipoarabinomannan and Determines Its Cell Envelope Localization to Control Phagolysosomal Fusion

**DOI:** 10.1371/journal.ppat.1004471

**Published:** 2014-10-30

**Authors:** Supriya Shukla, Edward T. Richardson, Jaffre J. Athman, Libin Shi, Pamela A. Wearsch, David McDonald, Niaz Banaei, W. Henry Boom, Mary Jackson, Clifford V. Harding

**Affiliations:** 1 Department of Pathology, Case Western Reserve University/University Hospitals Case Medical Center, Cleveland, Ohio, United States of America; 2 Mycobacteria Research Laboratories, Department of Microbiology, Immunology and Pathology, Colorado State University, Fort Collins, Colorado, United States of America; 3 Center for AIDS Research, Case Western Reserve University/University Hospitals Case Medical Center, Cleveland, Ohio, United States of America; 4 Department of Molecular Biology and Microbiology, Case Western Reserve University/University Hospitals Case Medical Center, Cleveland, Ohio, United States of America; 5 Department of Pathology, Stanford University School of Medicine, Palo Alto, California, United States of America; 6 Division of Infectious Diseases, Case Western Reserve University/University Hospitals Case Medical Center, Cleveland, Ohio, United States of America; Portland VA Medical Center, Oregon Health and Science University, United States of America

## Abstract

*Mycobacterium tuberculosis* (Mtb) virulence is decreased by genetic deletion of the lipoprotein LprG, but the function of LprG remains unclear. We report that LprG expressed in Mtb binds to lipoglycans, such as lipoarabinomannan (LAM), that mediate Mtb immune evasion. Lipoglycan binding to LprG was dependent on both insertion of lipoglycan acyl chains into a hydrophobic pocket on LprG and a novel contribution of lipoglycan polysaccharide components outside of this pocket. An *lprG* null mutant (Mtb Δ*lprG*) had lower levels of surface-exposed LAM, revealing a novel role for LprG in determining the distribution of components in the Mtb cell envelope. Furthermore, this mutant failed to inhibit phagosome-lysosome fusion, an immune evasion strategy mediated by LAM. We propose that LprG binding to LAM facilitates its transfer from the plasma membrane into the cell envelope, increasing surface-exposed LAM, enhancing cell envelope integrity, allowing inhibition of phagosome-lysosome fusion and enhancing Mtb survival in macrophages.

## Author Summary

The causative agent of tuberculosis, *Mycobacterium tuberculosis* (Mtb), persists in phagosomes inside infected macrophages. Mtb expresses lipoarabinomannan (LAM), which inhibits fusion of phagosomes with lysosomes as a means for Mtb to evade host defense. LAM is present in the cell envelope, which surrounds Mtb and interfaces with the host, but its localization remains unclear. We show that LprG, an Mtb lipoprotein, binds LAM and controls its distribution in the cell envelope. A mutant strain of Mtb that lacks LprG has less LAM at the surface of the cell envelope. This decreases LAM-mediated inhibition of phagosome-lysosome fusion, thereby impairing an immune evasion mechanism. We propose that LprG facilitates transfer of LAM from the plasma membrane into the cell envelope, enhancing its interaction with the host and ability to regulate host defense. Our results reveal mechanisms that determine bacterial cell envelope function and influence host-pathogen interactions and pathogen evasion of host defense.

## Introduction

Tuberculosis is the second leading cause of death from an infectious disease worldwide (http://www.who.int/mediacentre/factsheets/fs104/en/index.html). The causative agent, *Mycobacterium tuberculosis* (Mtb), is an intracellular bacterial pathogen that persists in phagosomes of infected macrophages. Mtb expresses a thick, waxy cell envelope of low permeability that contributes to antibiotic resistance and contains components (e.g. glycolipids, lipoglycans and lipoproteins) that play critical roles in regulating host responses and promoting survival of the pathogen [1]–[9]. Greater understanding of tuberculosis pathogenesis is needed as a foundation for development of drugs or vaccines to prevent or treat tuberculosis. Cell envelope components and the enzymes and transporters that control their synthesis and assembly serve as both determinants of pathogenesis and attractive targets for drug design.

The cell envelope of Mtb is rich in lipids and carbohydrates [1]–[3], [10], including lipoglycans such as lipoarabinomannan (LAM) and lipomannan (LM), and the glycolipid phosphatidyl-*myo*-inositolmannosides (PIMs). The PIMs include a core acylated glycolipid structure that is incorporated into the more highly glycosylated lipoglycans, LM and LAM, which are synthesized on the on the periplasmic face of the plasma membrane from PIM precursors by the elongation of mannan and arabinan chains [11]. LAM is essential for Mtb survival; the synthesis of LAM and cell envelope arabinans is targeted by the anti-mycobacterial agent ethambutol and DprE1 inhibitors currently under development [12]. LAM inhibits Mtb phagosome-lysosome fusion, providing a mechanism for Mtb evasion of host defense [13]–[18]. This effect is specific to ManLAM, the mannose capped LAM found in slow growing strains that are pathogenic in humans or other host species (e.g. Mtb, *M. bovis* BCG, *M. leprae*), and is not produced by phospho-*myo*-inositol-capped LAM (PI-LAM) found in *M. smegmatis* and *M. fortuitum* [13]. Recent studies have identified key steps in the biogenesis of LAM [1], [19], but the mechanisms by which LAM is assembled and organized into the cell envelope are unknown. A model for Mtb cell envelope structure is presented by Kaur et al [1].

Mtb expresses numerous, functionally diverse lipoproteins, many of which are contained in the cell envelope [20]–[22]. Mutation in lipoprotein processing by the disruption of lipoprotein signal peptidase *lspA* attenuates Mtb virulence [23], causes cell envelope permeability defects and increases sensitivity to antibiotics [24], suggesting potential roles of lipoproteins in maintaining Mtb cell envelope function. A recent proteomic study of the Mtb cell envelope identified LprG among the top 10 most abundant lipoproteins [25]. *LprG* (*Rv1411c*) forms an operon with *Rv1410c*, which encodes a putative efflux pump membrane protein, p55 [26]. Genetic deletion of this operon results in decreased virulence of Mtb in mice, and its deletion in *M. smegmatis* results in abnormal cell envelope morphology and permeability, suggesting its involvement in cell envelope biogenesis and/or maintenance of cell envelope integrity [26]–[30]. Despite evidence for the importance of LprG to Mtb virulence, its function remains uncertain.

Mtb LprG binds to LAM, LM and PIMs when expressed in *M. smegmatis*, and acyl moieties of these lipoglycans and glycolipids bind in a hydrophobic binding pocket of LprG [31]. Glycolipid binding was abrogated with a mutated version of LprG (LprG-V91W) with altered hydrophobic binding pocket structure [31]. In the studies reported here, we expressed acylated LprG in the native species, Mtb, to determine the function of LprG in Mtb. Surface plasmon resonance (SPR) binding assays were used to determine the kinetics and affinities for binding of different Mtb lipoglycans and glycolipids to LprG; the results revealed novel interactions between saccharide chains of LAM and LM and a site on LprG outside of the hydrophobic pocket, as well as interactions of the lipoglycan acyl chains with the LprG hydrophobic pocket. Furthermore, an LprG null mutant Mtb (Δ*lprG*) had reduced levels of surface-exposed LAM and decreased ability to inhibit Mtb phagosome-lysosome fusion, a LAM-dependent effect that is associated with Mtb survival and virulence. We propose that LprG binding to LAM facilitates the transfer of LAM from the plasma membrane (its site of synthesis) to the Mtb outer membrane. This leads to LAM expression at the cell surface, contributing to the ability of Mtb to inhibit phagosome-lysosome fusion and survive in macrophages. Our results highlight the importance of LprG and cell envelope components in the survival and virulence of Mtb and reveal a potentially important target for therapeutic intervention.

## Results

### LprG functions as a carrier of lipoglycans in Mtb

These studies explored for the first time the lipoglycan/glycolipid binding function of the lipoprotein LprG expressed in Mtb. Previous studies of LprG expressed in a fast-growing saprophytic mycobacterial species, *M. smegmatis*, had revealed a lipoglycan/glycolipid binding function [31], but the potential roles of LprG in lipoglycan biology in Mtb remained unexplored. The current studies provide a detailed understanding of lipoglycan/glycolipid interactions with LprG and reveal a novel role for LprG in determining the localization of LAM in the cell envelope.

**Figure 1:**
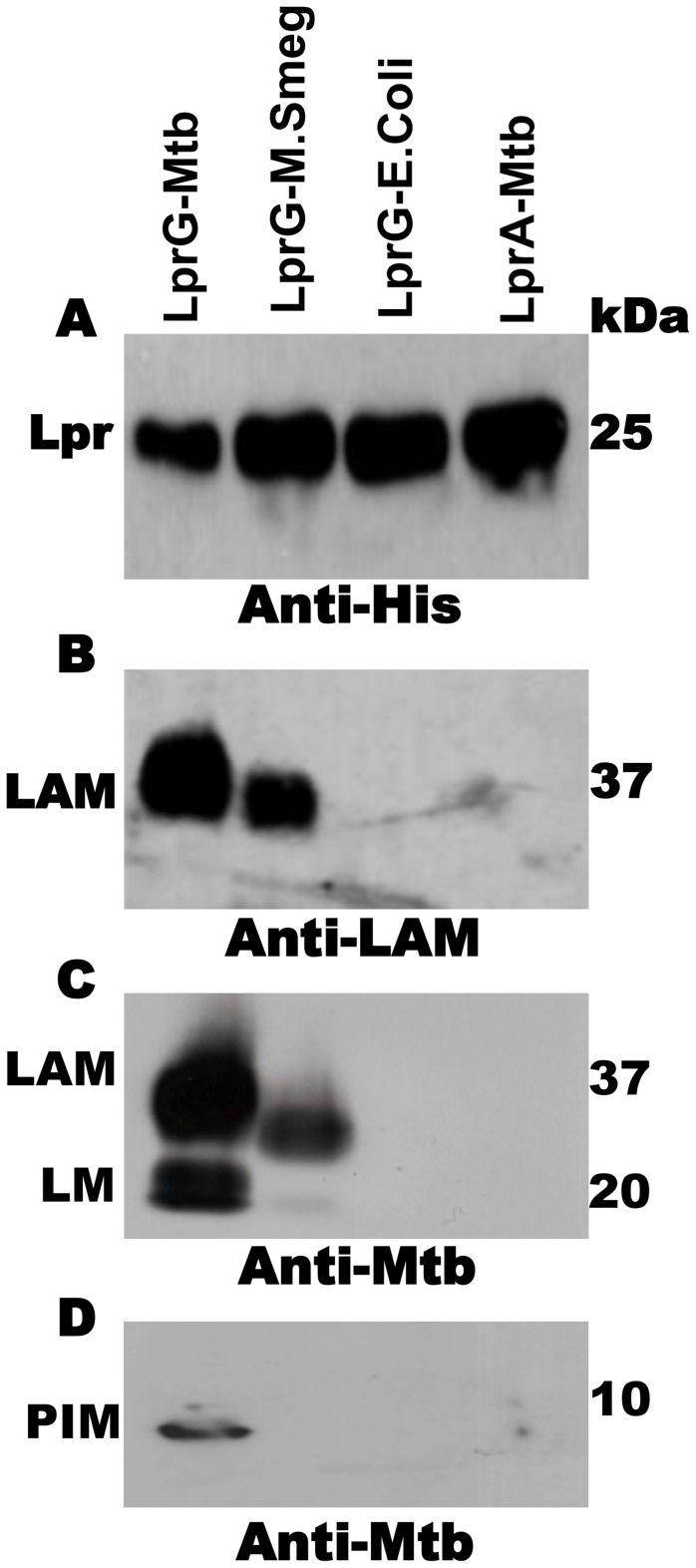
Acylated LprG binds lipoglycans in Mtb. SDS-PAGE analysis of proteins and co-purifying molecules isolated from Mtb H37Ra, *M. smegmatis* or *E. coli*. (**A**) Western blot with monoclonal anti-hexahistidine (anti-His_6_) to detect LprG or LprA. Mycobacterial components associated with lipoproteins were detected using (**B**) monoclonal anti-LAM antibody CS-35 or (**C and D**) rabbit polyclonal anti-Mtb antibody that detects both LAM and LM. Blots are representative of at least three independent experiments.

In order to study the association of lipoglycans and glycolipids with LprG in Mtb, the lipoprotein was expressed with a hexahistidine tag and isolated from Mtb lysate by Ni affinity and anion exchange chromatography. These studies used acylated LprG, rather than non-acylated LprG (NA-LprG), which was used in prior studies, as acylation state is likely to affect subcellular localization and intersection with specific glycolipid and lipoglycan species (acylation is linked with translocation across the plasma membrane). SDS-PAGE was used to analyze LprG and any co-purifying molecules. Silver stain and Western blot (Fig. 1A) with anti-hexahistidine antibody showed a single band at ∼25 kDa, corresponding to the approximate molecular weight of LprG. Western blot with CS-35 anti-LAM monoclonal antibody revealed a characteristic diffuse band at ∼37 kDa that was associated with acylated Mtb LprG expressed in both Mtb and *M. smegmatis* (Fig. 1B). A polyclonal anti-Mtb antibody that detects LAM, LM and PIMs revealed bands at the expected positions for these lipoglycans (∼37 kDa, ∼18 kDa and ∼10 kDa; corresponding to positions of these lipoglycan/glycolipid standards on the gel) in association with Mtb LprG expressed in Mtb and *M. smegmatis* but not *E. coli* (Fig. 1C and 1D). The detection of LAM was more intense than the detection of LM and PIMs, and the association of all three lipoglycans/glycolipids was greater when LprG was expressed in Mtb relative to *M. smegmatis* (Fig. 1B, C). These lipoglycans/glycolipids were not associated with Mtb-expressed acylated LprA, a lipoprotein that is homologous to LprG (hexahistidine-tagged LprA was expressed and purified using the same protocol as for LprG, see Materials and Methods); this control demonstrates the specificity of lipoglycan/glycolipid binding to LprG. These studies establish that acylated LprG binds LAM and LM in Mtb.

### SPR analysis of glycolipid and lipoglycan binding to LprG

While prior studies showed that binding of glycolipids to LprG involved insertion of glycolipid acyl chains into the LprG hydrophobic pocket (blocked by mutation of the hydrophobic pocket in LprG-V91W) [31], we hypothesized that lipoglycans with longer saccharide chains, e.g. LAM, might have additional functional interactions involving their saccharide moieties and other sites on LAM. To obtain insight regarding structures that determine lipoglycan/glycolipid binding to LprG, which may have implications for LprG function, we used SPR to measure the affinity and kinetics of LprG-substrate interactions and the effects of alterations in glycolipid/lipoglycan structures on binding to LprG. *In vitro* binding assays were performed using non-acylated LprG (NA-LprG) or NA-LprG-V91W, since the non-acylated versions retain glycolipid/lipoglycan binding properties and present technical advantages for expression, purification and use in binding assays (all references to LprG or LprG-V91W in SPR assays refer to the non-acylated versions). Expression of LprG or LprG-V91W in *E. coli* allowed purification of LprG without the presence of bound substrates (Fig. 1), facilitating subsequent glycolipid and lipoglycan binding assays. LprG and LprG-V91W were immobilized as ligands on sensor chips, and increasing concentrations of substrates were injected. Sensograms representing association and dissociation phases were obtained after subtracting non-specific binding to a blank sensor chip. The results provide the first determination of affinities for binding of different glycolipids and lipoglycans to LprG, allowing us to assess novel contributions of saccharide moieties to lipoglycan binding.

**Figure 2:**
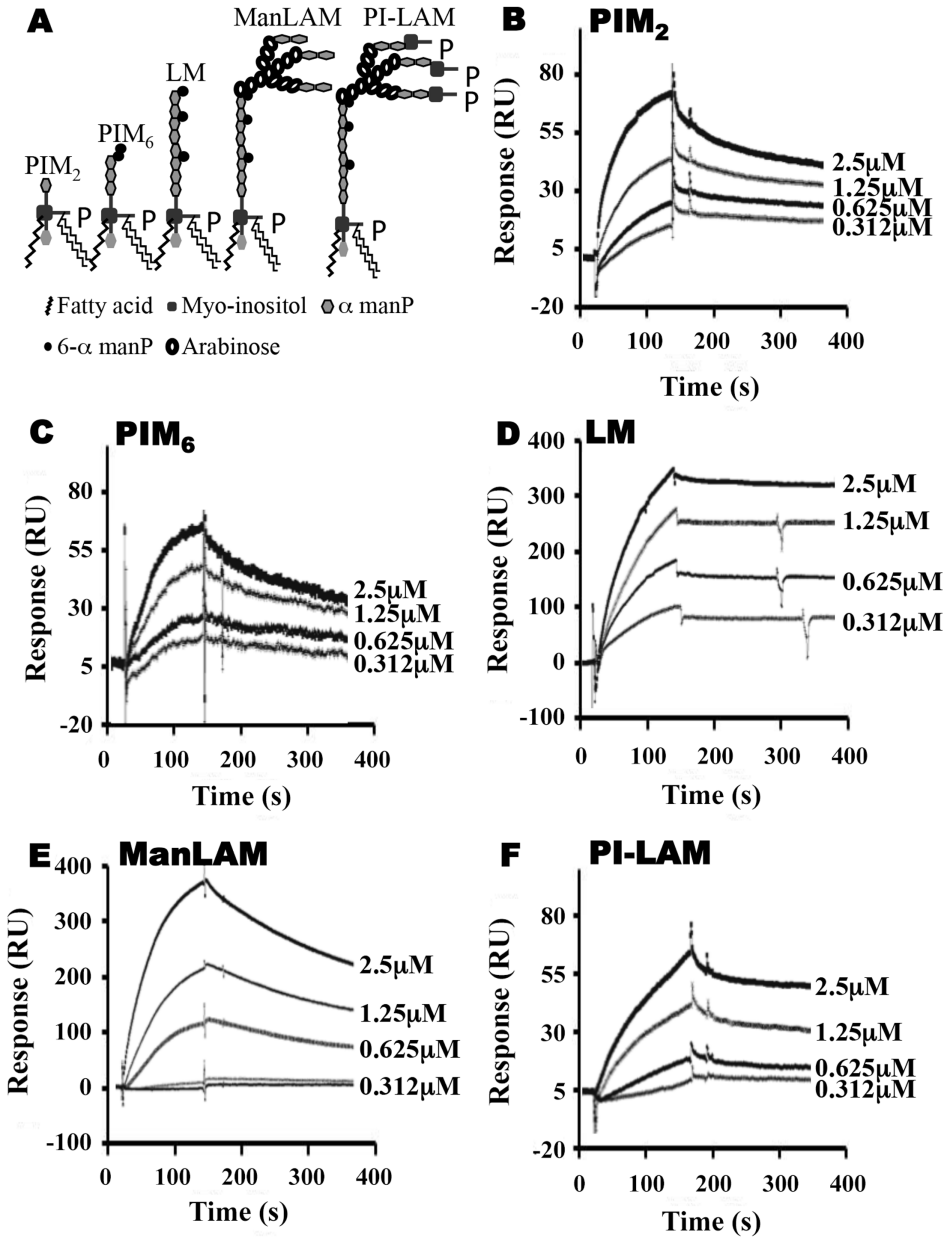
SPR analysis of substrate binding to LprG. (**A**) Schematic representation of mycobacterial glycolipids and lipoglycans used in this study. (**B–F**) Substrate binding to LprG was assessed by SPR. LprG was immobilized on a CM5 sensor chip. Sensograms were obtained by injecting increasing concentrations of (**B**) PIM_2_ (**C**) PIM_6_ (**D**) LM (**E**) ManLAM and (**F**) PI-LAM. Binding was measured as response units (RU). Binding curves were calculated with BIA evaluation 3.1 software with subtraction of non-specific binding of the substrates to the sensor chip control cells without immobilized LprG. Results are representative of three independent experiments.

To explore relationships between glycolipid structure and LprG-binding properties, we studied a panel of mycobacterial glycolipids and lipoglycans, including PIM_2_, PIM_6_, LM, ManLAM and PI-LAM, all of which share a mannosyl-phosphatidyl-myo-inositol domain with additional specific structural features (Fig. 2A). All of these molecules are expressed by Mtb except for PI-LAM, which is expressed by non-pathogenic mycobacteria. PIM_2_ (a precursor of PIM_6_, LM and LAM) has mannose residues at positions 2 and 6 of the *myo*-inositol ring of PI (Fig. 2A). PIM_2_ bound to LprG in a dose-dependent manner (Fig. 2B) but with relatively low affinity (K_D_ = 1.09×10^−6^ M, Table 1). PIM_6_, LM, ManLAM and PI-LAM all bound to LprG (Fig. 2C–F). Of these glycolipids, LM, which consists of a long linear mannan chain extending from position 6 of the *myo*-inositol ring, bound to LprG with the highest affinity (K_D_ = 1.58×10^−9^ M, Fig. 2D, Table 1). PIM_6_, which consists of a much shorter mannoside motif attached to position 6 of the *myo*-inositol ring, bound to LprG with the second highest affinity (K_D_ = 4.5×10^−8^ M, Fig. 2C, Table 1). LAM is generated from LM by the addition of a branched arabinan domain with species-specific terminal caps (ManLAM for Mtb, PI-LAM for non-pathogenic mycobacteria, Fig. 2A). ManLAM and PI-LAM bound to LprG with lower affinities than LM and PIM_6_ (K_D_ of 1.09×10^−7^ M for ManLAM and 1.6×10^−6^ M for PI-LAM, Fig. 2E–F, Table 1). Despite sharing the common acylated core structure that was previously implicated in binding to the hydrophobic pocket of LprG, these glycolipids and lipoglycans displayed distinct kinetics for LprG binding, indicating a previously unknown contribution of polysaccharide structures to LprG.

**Table 1:** Binding kinetics and affinities for interaction of Mtb substrates with LprG and LprG-V91W.

	K_on_ (1/MS)		K_off_ (1/S)		K_D_ (M)	
	LprG	LprG-V91W	LprG	LprG-V91W	LprG	LprG-V91W
PIM_2_	4.5e^3^	-	4.98e^−3^	-	1.09e^−6^	-
PIM_6_	219	662	9.8e^−6^	6.5e^−4^	4.5e^−8^	9.9e^−7^
LM	1.81e^3^	1.56e^3^	2.85e^−6^	3.09e^−4^	1.58e^−9^	1.98e^−7^
ManLAM	1.96e^3^	1.62e^3^	2.13e^−4^	2.13e^−3^	1.09e^−7^	1.31e^−6^
PI-LAM	599	-	9.8e^−4^	-	1.6e^−6^	-

**Figure 3:**
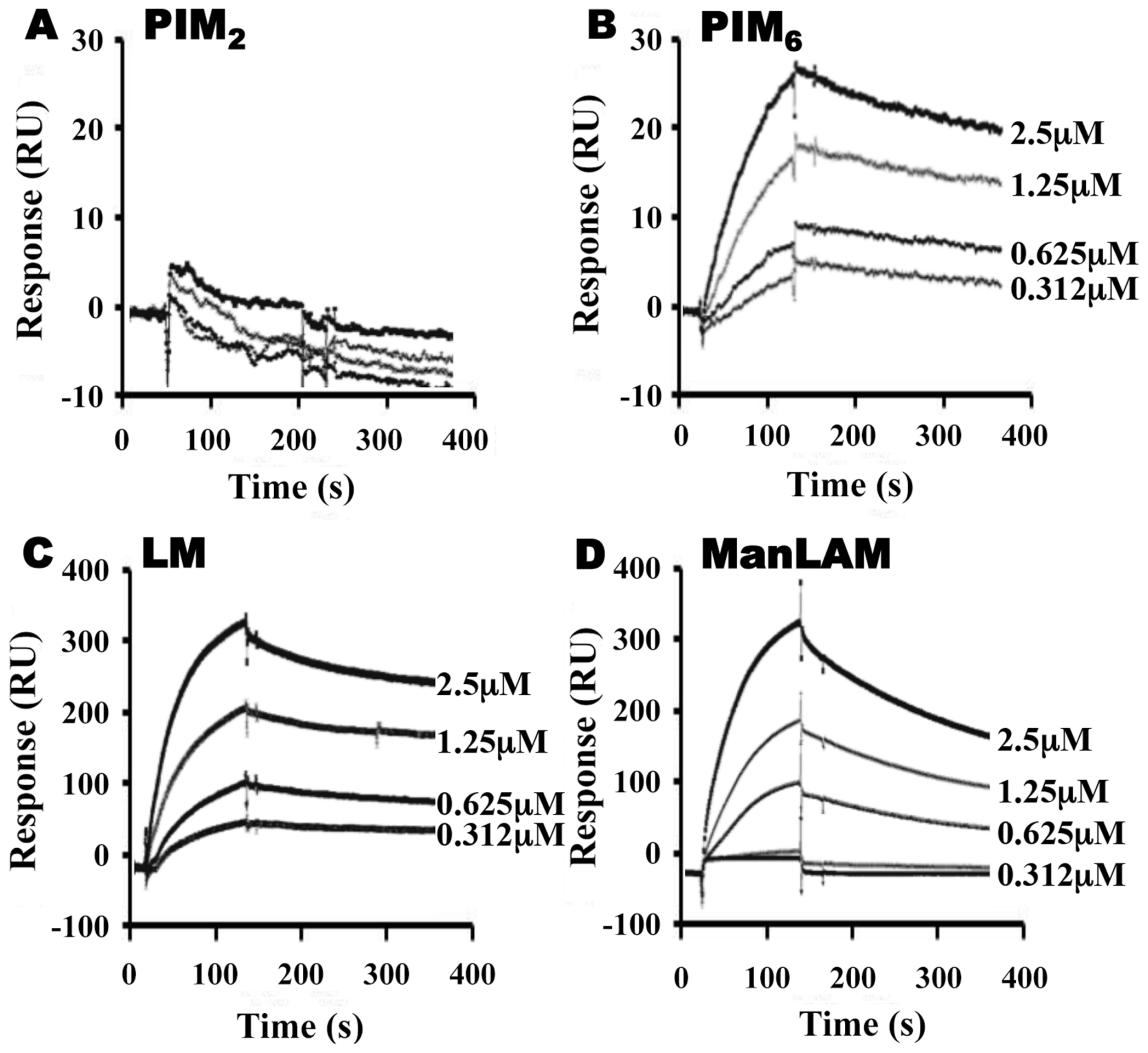
LprG V91W hydrophobic pocket mutation reduces binding of Mtb glycolipids and lipoglycans. LprG-V91W was immobilized on a CM5 sensor chip, and sensograms were obtained as in Fig. 2 for (**A**) PIM_2_ (**B**) PIM_6_ (**C**) LM and (**D**) ManLAM. Results are from one experiment and representative of three independent experiments.

To assess the relative contributions of the hydrophobic binding pocket vs. other sites on LprG, we studied glycolipid and lipoglycan binding to LprG-V91W, which has a mutated pocket that precludes binding of the acyl chains of triacylated glycolipids [31]. PIM_2_ (Fig. 3A) and PI-LAM failed to bind to LprG-V91W, but PIM_6_, LM and ManLAM bound to LprG-V91W with an affinity approximately 10- to 130-fold lower than their affinity for LprG (Fig. 3B–D) (Table 1). The loss of acyl-chain dependent interaction with LprG-V91W increased substrate dissociation rates (K_off_), indicating less stable interactions. These results indicate the presence of novel interactions of a subset of glycolipids/lipoglycans with LprG at a site outside of the hydrophobic pocket. Moreover, this suggests that these interactions include glycolipid and lipoglycan structures other than the acyl chains that bind within the hydrophobic pocket of LprG, consistent with the implication of novel contributions of saccharide structures of substrates in interactions with LprG.

**Figure 4:**
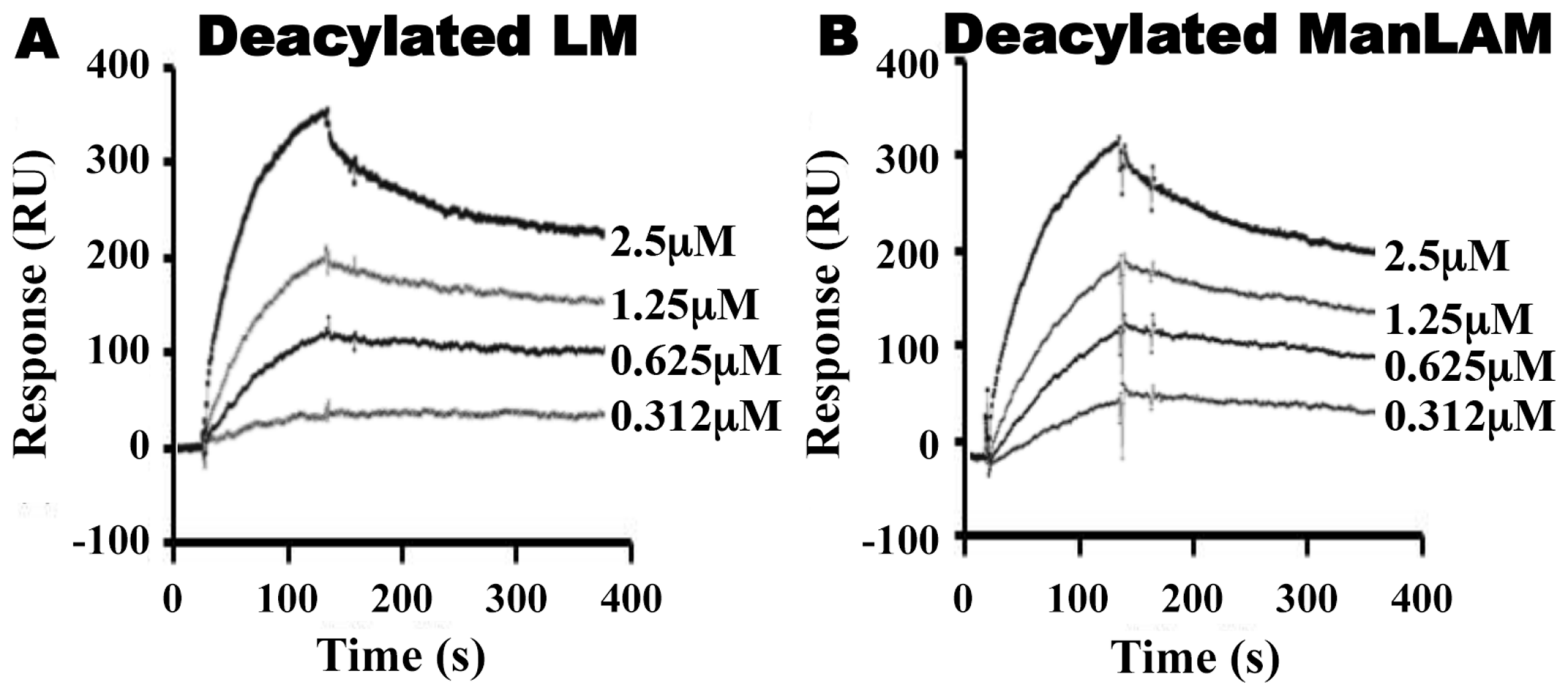
Deacylated ManLAM and LM retain binding to LprG at reduced levels. Binding to LprG was assessed as in Fig. 2 for (**A**) deacylated LM and (**B**) deacylated ManLAM. Results are from one experiment and representative of at least three independent experiments.

To further analyze the relative contributions of acyl and non-acyl structures of substrates to LprG binding, we subjected LM and ManLAM to alkaline deacylation. Deacylated LM and deacylated ManLAM both bound specifically to LprG (Fig. 4A and B, Table 2), and this binding was similar for LprG and LprG-V91W. This confirms that non-acyl structures of lipoglycans bind to a site on LprG different from the hydrophobic pocket. In support of this conclusion, loss of the acyl chains reduced the affinities of LM and ManLAM for LprG to values similar to the affinities of acylated LM and acylated ManLAM for LprG-V91W. Deacylation had little effect on the association rate for LM or ManLAM binding to LprG or LprG-V91W but resulted in increased dissociation rates with LprG. Overall, these results establish that Mtb lipoglycans and glycolipids bind to LprG via at least two different interactions at different sites on LprG, one being the acyl chain binding in the hydrophobic pocket and the other being interaction of saccharide chains (likely mannose chains) with LprG outside of the hydrophobic pocket. The ability of LprG to discriminate different glycolipids/lipoglycans based on differences in their saccharide structures may allow differential binding and release of different glycolipids/lipoglycans.

**Table 2:** Binding kinetics and affinities for interaction of Mtb deacylated substrates with LprG and LprG-V91W.

	K_on_ (1/MS)		K_off_ (1/S)		K_D_ (M)	
	LprG	LprG-V91W	LprG	LprG-V91W	LprG	LprG-V91W
Deacylated LM	1.56e^3^	1.73e^3^	3.09e^−4^	3.3e^−4^	1.98e^−7^	1.9e^−7^
Deacylated ManLAM	1.6e^3^	1.58e^3^	2.48e^−3^	2.28e^−3^	1.55e^−6^	1.44e^−6^

Values were calculated using BIAevaluation 3.1 software. Data shown are representative of at least three independent experiments.

### Mannose chains contribute to Mtb lipoglycan binding to LprG

**Figure 5:**
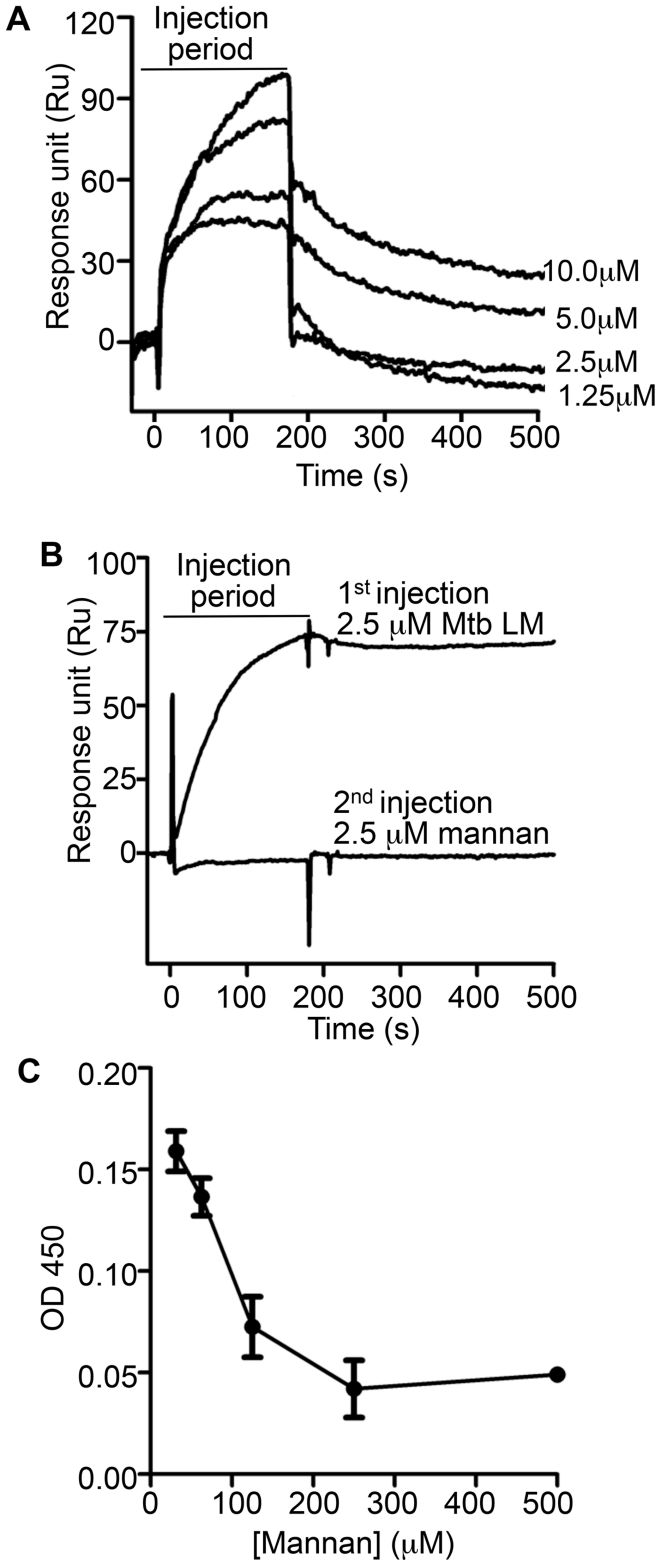
Mannan competition with Mtb lipoglycans for binding to LprG implicates a role for polysaccharide components of lipoglycans in LprG binding. (**A**) Binding of Mannan to LprG was assessed as in Fig. 2 at the indicated concentrations of mannan. (**B**) The ability of LM to compete with mannan for binding to LprG was assessed by SPR with sequential injection of LM (“1^st^ injection”) for 3 min followed by buffer for ∼10 min as the instrument prepared for injection of mannan (“2^nd^ injection”). This injection order was chosen due to the fast dissociation rate for mannan. The dissociation rate for LM (shown here) is slower than for LAM, but experiments with LAM provided qualitatively similar results. Results for mannan binding in panels A and B are directly comparable except for the absence (panel A) or presence (panel B) of prior LM injection. Results for panels A and B are representative of two independent experiments. (**C**) Mannan inhibited LAM binding to LprG in a solid phase competitive binding assay. LprG was incubated with mannan at the indicated concentrations for 30 min and added to wells of plates coated with ManLAM. After incubation and washing, the plates were incubated with anti-LprG antibody followed by an HRP-linked secondary antibody to detect LprG binding to ManLAM (see Materials and Methods). Data are expressed as the means from three independent experiments.

To investigate whether mannan components of LAM and LM contribute to lipoglycan binding to LprG, we tested the ability of *S. cerevisiae* mannan to bind to LprG. *S. cerevisiae* mannan was selected since its structure is comparable to the polysaccharide components of Mtb lipoglycans, except for the absence of the phosphatidyl-*myo*-inositol lipid anchor present in Mtb lipoglycans. SPR assays revealed mannan binding to LprG in a dose dependent manner, although with a lower affinity compared to LAM and LM (K_D_ = 8.8×10^−5^ M, Fig. 5A and Table 3, compare to Table 1). Next, to test whether mannan and Mtb lipoglycans have overlapping binding site(s) on LprG, we assessed their ability to compete for binding to LprG in SPR assays. The potential approach to saturate immobilized LprG with mannan in a first injection and subsequently inject LAM or LM was not feasible due to the rapid dissociation rate of mannan (4.5×10^−2^ sec^−1^), which allowed dissociation of pre-bound mannan before injection of LAM or LM could be completed. Accordingly, we first injected the lipoglycan (e.g. LM, 2.5 µM), which bound to LprG (Fig. 5B, “first injection”); a subsequent injection of mannan (2.5 µM; Fig. 5B, “second injection”) did not reveal mannan binding in contrast to the ability of mannan to bind to LprG in the absence of LM (compare Fig. 5A and 5B). Similar results were obtained with LAM and mannan. These results indicate that mannan and Mtb lipoglycans compete for binding to LprG. To confirm this conclusion with a different approach, we used a solid phase binding assay that measured LprG binding to plate-immobilized ManLAM. LprG bound to plate-immobilized ManLAM in a dose-dependent fashion. When LprG was incubated with mannan, however, the binding of LprG to ManLAM was inhibited by the presence of mannan in a dose-dependent manner (Fig. 5C). These data further indicate that mannan and Mtb lipoglycans compete for binding to LprG. Taken together, these results support our other evidence that mannose residues contribute to the binding of Mtb lipoglycans to LprG, in addition to contributions of acyl chain interactions.

**Table 3:** Binding kinetics and affinity for interaction of *Saccharomyces cerevisiae* mannan with LprG.

	K_on_ (1/MS)	K_off_ (1/S)	K_D_ (M)
Mannan	4.0e^−4^	4.5e^−2^	8.8e^−5^

### Deletion of *lprG* results in decreased levels of detectable LAM at the bacterial surface

We hypothesized that LprG may serve a function in the synthesis of LAM (and possibly LM and PIM) or their assembly into the cell envelope (possibly mediating their transfer from anchorage in the plasma membrane to allow localization at sites more peripheral in the cell envelope). This hypothesis suggests that deletion of LprG might alter the expression or localization of these molecules and influence the cell envelope properties of Mtb. Accordingly, we studied the effects of LprG expression on Mtb cell envelope properties using Mtb H37Ra Δ*lprG* and Mtb H37Rv Δ*lprG* strains with deletion of the *lprG* gene, and H37Rv Δ*lprG::lprG-Rv1410c*, a complemented version of H37Rv Δ*lprG* expressing the operon encoding LprG and p55.

To assess whether deletion of *lprG* affects the expression of the glycolipids to which LprG binds, e.g. LAM, we used Western blotting to assess the total bacterial expression of LAM and flow cytometry to detect the expression of LAM at the bacterial cell surface. Whole cell lysates of Mtb H37Ra and H37Ra Δ*lprG* were analyzed by SDS-PAGE and Western blotting with anti-LprG, which confirmed the absence of LprG in Mtb H37Ra Δ*lprG* (Fig. S1A). Western blotting with anti-LAM monoclonal antibody CS-35 or anti-Mtb polyclonal antibody (Fig. S1B, C) and detection of glycolipids and lipoglycans with carbohydrate staining (Fig. S1D) showed that Mtb H37Ra and H37Ra Δ*lprG* had similar total cellular expression of LAM and LM. Assessment of lipoglycan and glycolipid expression in Mtb H37Rv, H37Rv Δ*lprG* and H37Rv Δ*lprG::lprG-Rv1410c* strains by SDS-PAGE analysis, thin layer chromatography and periodic acid/Schiff staining revealed comparable amounts of LAM, LM and PIMs in all three strains.

**Figure 6:**
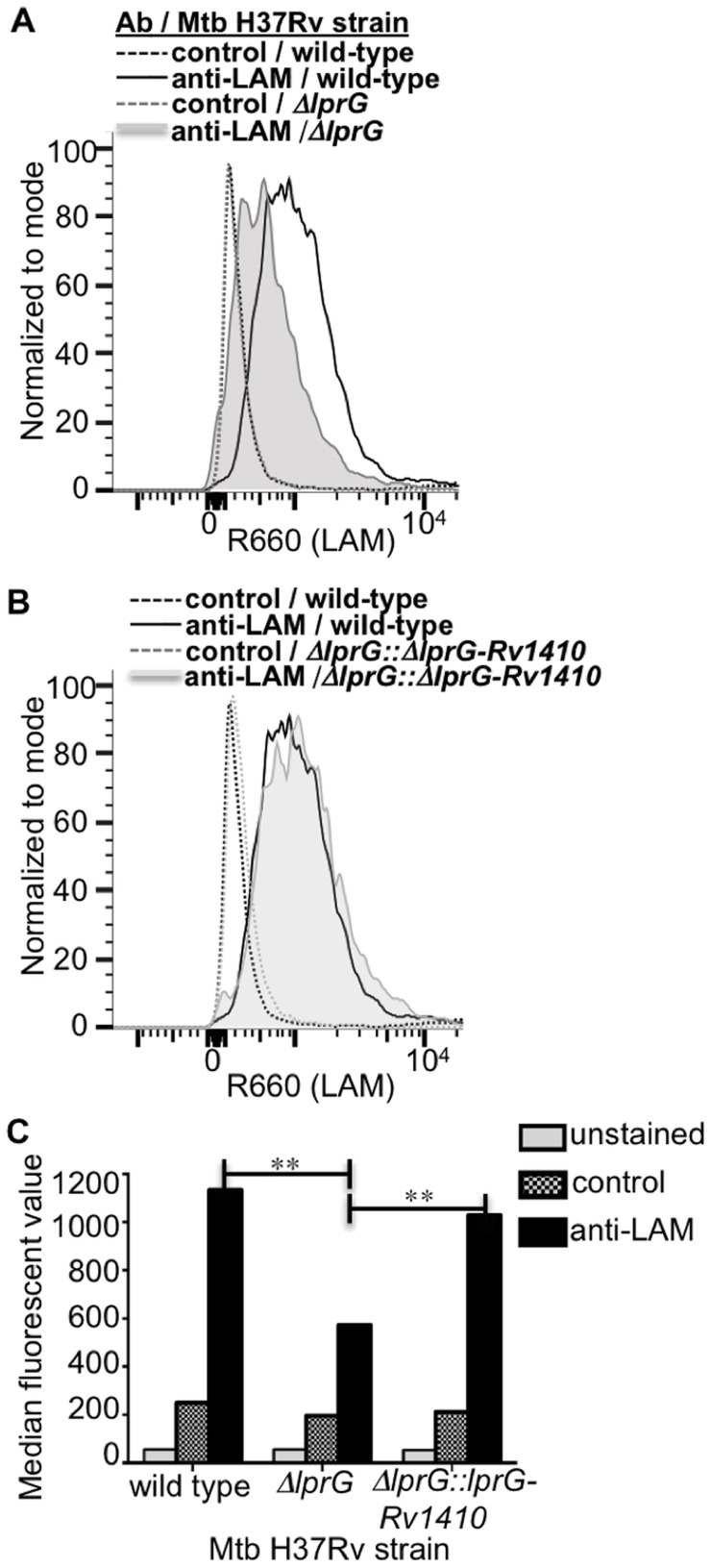
Deletion of *lprG* reduces LAM expression on the Mtb cell surface. Cultures of H37Rv, H37Rv Δ*lprG* and H37Rv Δ*lprG*::*lprG-Rv1410c* were seeded at a starting density of 0.05 OD_600_ and harvested after 1 week. Bacteria were stained with rabbit anti-ManLAM antiserum or control normal rabbit serum and analyzed by flow cytometry. (**A, B**) Histograms showing LAM surface staining of H37Rv, H37Rv Δ*lprG* and H37Rv Δ*lprG*::*lprG-Rv1410c*. (**C**) Median fluorescence values (MFVs) from panel A. **P<0.001. Specific MFV was defined as MFV with anti-LAM minus MFV with control serum. Specific MFVs were 900 for H37Rv, 360 for H37Rv Δ*lprG* and 870 for H37Rv Δ*lprG*::*lprG-Rv1410c*. Results are representative of two independent experiments.

Since deletion of *lprG* did not affect overall expression of LAM in Mtb, we assessed the hypothesis that LprG may affect the distribution of LAM in Mtb. Specifically, we considered that LprG may affect LAM assembly into the cell envelope, possibly by mediating removal of LAM from anchorage in the plasma membrane to allow its localization to sites more peripheral in the cell envelope. To determine whether the deletion of *lprG* reduces the amount of LAM detected at the bacterial cell surface, Mtb H37Rv, the knockout strain H37Rv Δ*lprG*, and the complemented control strain H37Rv Δ*lprG::lprG-Rv1410c* were stained with rabbit anti-ManLAM anti-serum or control normal rabbit serum and analyzed by flow cytometry. Specific LAM staining was >2-fold higher on Mtb H37Rv relative to Mtb H37Rv Δ*lprG*, and 1.8-fold higher on Mtb H37Rv Δ*lprG::lprG-Rv1410c* relative to Mtb H37Rv Δ*lprG* (Fig. 6). Furthermore, similar results were obtained with Mtb H37Ra (specific LAM staining was 7-fold higher on Mtb H37Ra relative to Mtb H37Ra Δ*lprG*). In summary, deletion of *lprG* did not affect total bacterial expression of LAM but did alter LAM distribution to substantially decrease its cell surface exposure.

### Deletion of *lprG* causes increased Mtb phagosome-lysosome fusion

**Figure 7:**
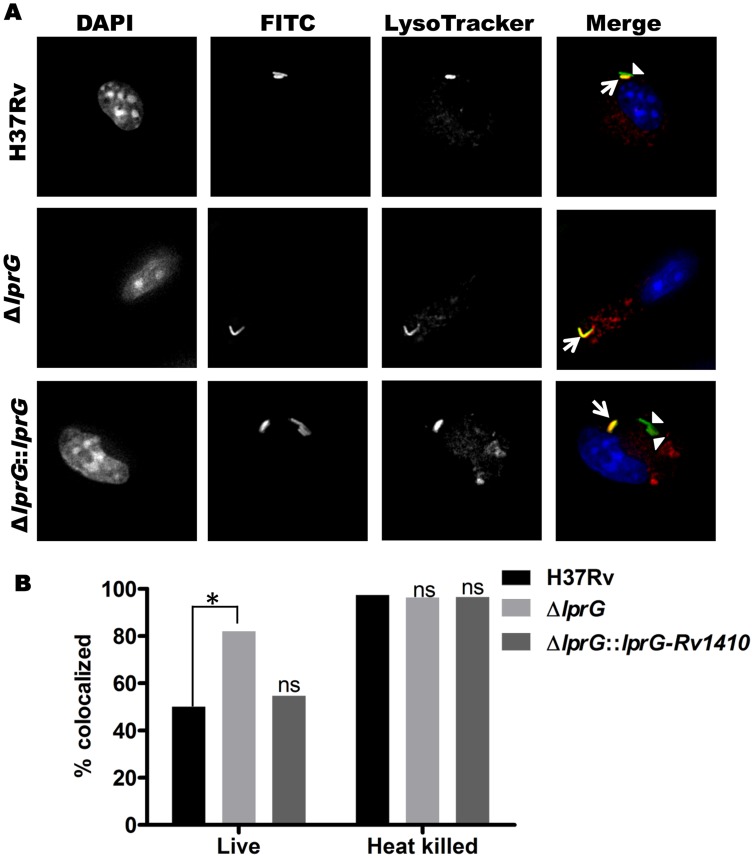
Genetic deletion of LprG reduces the ability of Mtb to inhibit phagosome-lysosome fusion. Murine bone marrow-derived macrophages were incubated with LysoTracker Red to label lysosomes and incubated with FITC-labeled Mtb for 60 min (30 min pulse+30 min chase). (**A**) Representative photomicrographs of macrophages after incubation with Mtb H37Rv, H37Rv Δ*lprG* and H37Rv Δ*lprG*::*lprG-Rv1410c*. Signals are rendered in grey scale in the first three columns, which show signal from a single label. In the fourth column overlay images (“Merge”, in color), Mtb H37Rv Δ*lprG* shows increased co-localization with lysosomal marker relative to Mtb H37Rv and H37Rv Δ*lprG*::*lprG-Rv1410c*. Arrowheads indicate FITC-labeled Mtb contained within phagosomes not colocalized with lysosomes (green); arrows indicate Mtb phagosome-lysosome co-localization (yellow). The p values were calculated using Fisher's exact test. (**B**) Percent co-localization of Mtb phagosomes with lysosomes (*, p = 0.0002; ns, not significant relative to Mtb H37Rv). Heat-killed bacteria are known to lack the ability to inhibit phagosome-lysosome fusion and were used as a positive control. Results shown here are from one experiment and are representative of at least two independent experiments.

Bacterial phagosomes fuse with lysosomes to produce phagolysosomes, which can mediate killing of some bacteria. Inhibition of phagosome-lysosome fusion is one means by which Mtb evades host defenses, and LAM is known to inhibit phagosome-lysosome fusion (i.e. inhibit Mtb phagosome maturation) [13]–[15]. We hypothesized that decreased surface expression of LAM in Mtb Δ*lprG* would decrease the ability of this Mtb strain to inhibit phagosome maturation. To test this hypothesis, murine bone marrow-derived macrophages were incubated with LysoTracker Red to label lysosomes and then infected with FITC-labeled Mtb H37Rv, H37Rv Δ*lprG* or H37Rv Δ*lprG::lprG-Rv1410c*. Mtb phagosome-lysosome fusion was assessed by co-localization of LysoTracker Red with intracellular FITC-Mtb (within DAPI-stained cells) as assessed by fluorescence microscopy (Fig. 7A). After 1 h of infection, Mtb H37Rv demonstrated 50% co-localization with LysoTracker Red, whereas Mtb H37Rv Δ*lprG* showed 82% co-localization (p = 0.0002). Mtb H37Rv Δ*lprG::lprG-Rv1410c* showed reversion to the wild-type phenotype with 55% lysosomal co-localization (not significantly different from Mtb H37Rv, p = 0.6181) (Fig. 7B). As a positive control for uninhibited phagosome-lysosome fusion, heat-killed Mtb of all three strains proceeded to near complete phagosome-lysosome fusion (96–97% co-localization of Mtb and lysosomal markers), consistent with prior observations that heat-killed Mtb does not inhibit phagosome maturation [16], [17]. In conclusion, deletion of *lprG* reduces the availability of LAM for interactions with the host cell machinery that produce inhibition of phagosome-lysosome fusion. These results and the observation that deletion of *lprG* reduced cell-surface expression of LAM by Mtb both indicate that LprG controls the localization of LAM and suggest that LprG has a role in the insertion or assembly of lipoglycans into the Mtb cell envelope, perhaps by mediating its removal from the plasma membrane to allow localization to more peripheral sites in the cell envelope. The ability of LprG to affect the expression of LAM on the Mtb cell surface has important implications, as this mechanism determines the access of LAM to interaction with host cells.

## Discussion

The studies reported here explore the importance of determinants of cell envelope architecture in host-pathogen interactions and pathogenesis of bacterial infections. In particular, we shed new insight into the role of LprG expression in the pathogenic species, Mtb. Our data reveal novel structure-function relationships that determine glycolipid binding by LprG, including aspects of LAM binding that are specific to the LAM species expressed by Mtb (ManLAM, as opposed to PI-LAM expressed by non-pathogenic mycobacteria). Specifically, we demonstrate that LprG expressed in Mtb binds to ManLAM, a major immunomodulatory molecule of the Mtb cell envelope. Differences in the kinetics and affinities of LprG binding by ManLAM, PI-LAM, LM, PIM_2_, PIM_6_ and deacylated versions of LM and LAM reveal novel contributions of the saccharide moieties of these substrates to LprG binding. ManLAM, PI-LAM, LM, PIM_2_ and PIM_6_ all share the core acylated PIM structure, allowing their acyl chains to bind in the LprG hydrophobic pocket, as indicated by reductions in binding of all of these species to pocket-mutated LprG V91W relative to LprG (Table 1). The V91W mutation reduced the binding affinity by ∼10–100-fold for ManLAM, LM, and PIM_6_, and reduced binding to undetectable levels for PIM_2_ or PI-LAM. However, variation in the saccharide moieties of PIM, LM and LAM also influenced binding to LprG, revealing novel binding interactions of saccharide components at an additional site outside of the hydrophobic pocket (which persist with the LprG V91W pocket mutant). Deacylated ManLAM and deacylated LM were found to bind LprG, albeit with lower affinity than the acylated versions, and their binding was not substantially affected by the V91W pocket mutation. This further establishes a contribution of the substrate saccharide moieties to binding at a site outside of the LprG hydrophobic pocket. PIM_2_ lacks the longer mannan chain extensions from the PIM core structure that are seen in the other glycolipids, and its lower affinity for LprG binding may reflect lack of mannan interactions with LprG. Variation of the terminal capping of LAM may also affect LprG binding, given the reduced affinity of PI-LAM (which lacks terminal mannose residues) relative to ManLAM.

In summary, these results indicate that binding interactions between Mtb lipoglycans and LprG extend beyond the lipoglycan acyl chains and implicate a role for saccharide chains of Mtb lipoglycans in binding to LprG. First, deacylated Mtb lipoglycans bind to LprG (albeit at lower affinity than acylated lipoglycans). Second, acylated lipoglycans bind to LprG even when the hydrophobic pocket where the acyl chains bind is mutated to prevent their interaction (albeit with lower affinity than to wild-type LprG). Notably, the affinity of binding of the deacylated lipoglycans was not affected by mutation of the hydrophobic pocket (Table 2). Interestingly, in these two situations (acylated lipoglycan binding to pocket-mutated LprG vs. deacylated lipoglycan binding to LprG), whereby different approaches the contribution of acyl chains to LprG binding has been removed and the remaining interactions are implicated to be with saccharide chains, the binding affinities are very similar, consistent with our model (Tables 1 and 2, compare K_D_ for binding of acylated vs. deacylated LAM to LprG vs. LprG-V91W, or compare K_D_ for binding of acylated vs. deacylated LM to LprG vs. LprG-V91W).

The discovery of contributions of lipoglycan polysaccharide moieties to LprG binding suggested that these interactions involve lipoglycan mannan residues, since this property is shared by lipoglycans with mannose saccharides (e.g. LAM) and that lack other saccharide components (e.g. arabinan) found in LAM. Furthermore, we directly tested the contribution of mannan structures to LprG binding by use of *S. cerevisiae* mannan, which has structural similarity to the mannan chains of Mtb LM and LAM and also binds to LprG, although with a lower affinity. In competition binding assays, LM blocked mannan binding to LprG (Fig. 5B), and mannan inhibited LAM binding to LprG (Fig. 5C). These two findings further support the hypothesis that mannan and the Mtb lipoglycans (LAM, LM) compete for a binding site on LprG and further support the hypothesis that mannan chains of these Mtb lipoglycans contribute to their binding to LprG. In summary, although saccharide interactions are not essential for low affinity glycolipid binding to LprG, mannan chains interact with LprG to enhance substrate-LprG binding. We propose a model for lipoglycan binding to LprG that involves both acyl chain binding in the LprG hydrophobic pocket and mannan chain interactions with LprG outside of the pocket.

In addition to determining the structural basis for LprG-glycolipid interactions, we assessed the functional contributions of LprG using genetic deletion models. Although *lprG* deletion did not alter the total cellular expression of LAM, LM and PIM by Mtb, our results reveal the striking discovery that LprG controls expression of LAM at the cell surface of Mtb, presumably via its lipoglycan binding function. Thus, LprG influences the spatial organization of LAM within the Mtb cell envelope, perhaps via a role in LAM insertion or assembly into the cell envelope. One possibility is that LprG mediates the removal of lipoglycans from acyl-chain anchorage in the plasma membrane to allow their localization to more peripheral sites in the cell envelope.

Altered localization of LAM in the cell envelope may have significant implications for cell envelope functions and host-pathogen interactions. Deletion of LprG affects general measures of cell envelope integrity (e.g. malachite green decolorization, Congo red binding), consistent with other studies that associated altered cell envelope permeability with deletion of the *lprG-Rv1410c* operon [28], [30], [32]. Since LAM is implicated in inhibiting the fusion of Mtb phagosomes with lysosomes [13]–[15], we tested the hypothesis that reduced expression of surface-exposed LAM in the Mtb cell envelope would decrease LAM interactions with host cells and limit inhibition of phagosome maturation in Mtb H37Rv Δ*lprG*. Consistent with our hypothesis, we observed decreased inhibition of Mtb phagosome-lysosome fusion with Mtb H37Rv Δ*lprG* relative to wild-type Mtb H37Rv. Thus, deletion of *lprG* reduced the ability of Mtb to inhibit phagosome maturation, consistent with the known role for LAM in inhibiting phagosome maturation and our discovery that deletion of *lprG* diminishes expression of LAM at the bacterial cell surface, which we surmise diminishes its availability to interact with host cells.

In summary, our results reveal novel structural determinants of the binding of PIMs, LM and LAM to LprG and a novel role for LprG in determining LAM distribution in the cell envelope. While LprG is not necessary for LAM biosynthesis, it controls the expression of LAM at the bacterial cell surface, perhaps via a role in transferring LAM from the plasma membrane for localization in the cell envelope. Thus, LprG determines the accessibility of LAM for interactions with host cells to regulate phagosome-lysosome fusion and other host-pathogen interactions. These findings have important implications for fundamental determinants of host-pathogen interactions during infection with Mtb. LprG clearly influences Mtb phagosome maturation; it is required for optimum inhibition of phagosome maturation by Mtb, and it may thereby contribute to immune evasion by this mechanism. The influence of LprG on LAM localization in the Mtb cell envelope may affect the integrity of the cell envelope and its effectiveness as a permeability barrier, which is critical to Mtb survival within the host. Findings in this study provide novel insight into mechanisms of pathogenesis and host resistance to Mtb infection, and they suggest the possibility that pharmacologic disruption of LprG expression or function might result in a leaky cell envelope (making Mtb less resistant to drugs and antibiotics) and reduced ability of Mtb to evade host defense mechanisms by inhibiting phagosome-lysosome fusion. In conclusion, LprG is a critical determinant of Mtb virulence and host-pathogen interactions during Mtb infection and may be used as a potentially important target for therapeutic intervention.

## Materials and Methods

### Ethics statement

The Institutional Animal Care and Use Committee of Case Western Reserve University approved all animal studies (protocol 2012–0007). Studies were performed in accordance with recommendations of the Guide for the Care and Use of Laboratory Animals of the National Institutes of Health.

### Mtb strains and preparation of bacterial lysates

Mtb strains H37Rv and H37Ra were obtained from American Type Culture Collection (Manassas, VA). LprG null strains were generated from Mtb H37Rv (H37Rv Δ*lprG*; N. Banaei) and H37Ra (H37Ra Δ*lprG*; E.T. Richardson) using a specialized transducing phage targeting 571 bp within the *lprG* gene locus for homologous recombination with a hygromycin resistance cassette. Mtb H37Rv Δ*lprG::lprG-Rv1410c* was then generated by complementing Mtb H37Rv Δ*lprG* with the native Rv1411c/1410c operon expressed off the kanamycin-selective integrating plasmid pMV306. Bacteria were cultured in Middlebrook 7H9 broth (Difco, Lawrence, KS) supplemented with 10% albumin/dextrose/catalase (BD, Franklin Lakes, NJ), 0.05% Tween 80 and 0.2% glycerol plus 100 µg/ml hygromycin B (Invivogen, San Diego, CA) for H37Ra Δ*lprG* and H37Rv Δ*lprG* or 50 µg/ml kanamycin (Sigma, St. Louis, MO) for H37Rv Δ*lprG::lprG-Rv1410c*.

To assess bacterial expression of lipoglycans, lysates were prepared from Mtb H37Ra and H37Ra Δ*lprG*. Bacteria were grown to mid log phase in 50 ml of Sauton's medium, pelleted, suspended in PBS, and sonicated in an ice bath for 40 min in 10-second cycles at 50 W in a Misonix sonicator (Misonix Inc., Farmingdale, NY). Insoluble debris was pelleted at 2000× *g* for 5 min, and supernatants were stored at −80°C. Samples were analyzed by SDS-PAGE as described below.

### Cloning, expression and purification of hexahistidine-tagged proteins

Constructs for expression of recombinant lipoproteins LprG, LprA, non-acylated LprG (NA-LprG) and non-acylated LprG-V91W (NA-LprG-V91W) were cloned previously [6], [31]. LprG, LprA, NA-LprG and NA-LprG-V91W were expressed in wild-type Mtb H37Ra, *M. smegmatis* or *E. coli* and purified as described [6], [31]. For expression in Mtb H37Ra, constructs were digested with NdeI and HindIII (New England Biolabs, Ipswich, MA) and ligated into the shuttle vector pVV16 (provided by J. Belisle, Colorado State University, Fort Collins, CO) under control of the constitutively active hsp60 promoter and in-frame with a C-terminal hexahistidine tag. Mtb was transformed by electroporation with a Gene Pulser (Bio-Rad, Hercules, CA) set at 2.5 kV, 25 µF, and 800 ohms. Mtb was grown with kanamycin selection (50 µg/ml) to late log phase, isolated by centrifugation at 6000 *g* for 20 min at 4°C, and suspended for 15 min at 37°C in lysis buffer. Bacteria were disrupted by four passages through a French press (2000 psi). Insoluble material was removed by ultracentrifugation at 100,000 *g* for 1 h at 4°C. The supernatant was incubated for 2–4 h at 4°C with Ni beads (Qiagen, Valencia, CA), which were then washed 3 times with 25 volumes of wash buffer (50 mM sodium phosphate, 1 M NaCl, 20 mM imidazole, 10% glycerol, pH 8.0). Bound protein was dissociated with elution buffer consisting of 50 mM sodium phosphate, 300 mM NaCl, 450 mM imidazole, pH 8.0. Samples were exchanged into 20 mM Tris, pH 8.0 using PD-10 columns (GE Healthcare, Uppsala, Sweden) and further purified by anion-exchange chromatography. Recombinant LprG was bound to HiTrap Q FF columns (GE Healthcare) and eluted by stepwise addition of 50, 150, 200 and 1000 mM NaCl. Recombinant LprG eluted with 50–200 mM NaCl was collected and concentrated using 10-kDa cutoff Centricon units (Amicon, Billerica, MA). Protein yields were determined by BCA protein assay (Pierce, Rockford, IL).

### SDS-PAGE and western blots

Protein preparations or bacterial lysates were electrophoresed on 12% or 4–20% Mini-PROTEAN TBX Precast SDS-PAGE gels (BioRad, Hercules, CA). Protein purity was analyzed with Silver Stain Plus (BioRad). For Western blot analysis, materials were transferred to polyvinylidene difluoride membranes (Millipore), which were blocked with 5% milk in PBS supplemented with 0.1% Tween-20 (PBST) for 1 h at room temperature. Membranes were incubated overnight at 4°C with mouse monoclonal anti-hexahistidine antibody (Santa Cruz Biotechnology, Santa Cruz, CA), rabbit polyclonal anti-Mtb antibody (GENway, Hayward, CA), mouse monoclonal anti-LAM antibody (CS-35) and mouse monoclonal anti-LprG antibody (clone α–Rv1411c, NR-13806, NIH Biodefense and Emerging Infections Research Resources Repository; BEI, Manassas, VA). Blots were washed three times with PBST, incubated for 1–2 h at room temperature with secondary antibodies conjugated to horseradish peroxidase, washed three times with PBST and visualized using a chemiluminescence kit (Pierce). Alternatively, membranes were stained to detect carbohydrate with the Glycoprotein Staining Kit (Pierce, Rockford, IL).

### Preparation of deacylated ManLAM and LM

Purified ManLAM and LM from Mtb were deacylated by treatment of 1 mg ManLAM or LM with 0.1 N NaOH for 2 h at 37°C. The reaction mixture was neutralized with 10% acetic acid to pH ∼8.0, and dialyzed against distilled water for one day at room temperature. The sample was dried, loaded onto a Bio-gel P100 column and eluted with 0.1 N acetic acid. Fractions were collected and analyzed by SDS-PAGE to confirm loss of migration due to deacylation. Fractions containing deacylated ManLAM or LM were pooled, dialyzed against distilled water and dried. Sample purity was assessed by gas chromatography-mass spectrometry.

### Surface plasmon resonance (SPR) binding assay

SPR binding experiments were performed on a BIAcore 3000 instrument using CM5 (carboxymethylated) sensor chips and HBSN running buffer (10 mM HEPES and 150 mM NaCl, pH 7.4) (GE Healthcare, Uppsala, Sweden). Non-acylated (NA)-LprG was used for SPR studies and purified from *E. coli* to prevent prior occupancy of LprG binding site(s) by Mtb glycolipids and lipoglycans that might hinder the binding of exogenous purified LprG ligands used in SPR analyses. Our previous studies [31] reveal that NA-LprG retains its ability to bind to Mtb glycolipids and lipoglycans, and a similar spectrum of LprG-associated molecules was found associated with acylated LprG expressed in Mtb (Fig. 1). In addition, it is unlikely that the acyl chains of LprG would interact with residues at or near to the lipoglycan binding pocket, as the N-terminal site of acylation of LprG is ∼50 Å away from the lipoglycan pocket (discussed by Drage et al [31]). Acylated LprG (Fig. 1) and NA-LprG (Figs. 2–5) both bind LAM, LM and PIMs.

NA-LprG and NA-LprG-V91W ligands were immobilized on CM5 sensor chips by amine coupling [33]. A 1∶1 mixture of 0.1 M n-hydroxysuccinimide (NHS) and 0.1 M ethylene diamine (GE Healthcare) was injected to activate carboxyl groups on the CM5 matrix, and 30 ml of a 40 mg/ml solution of ligand in 10 mM acetate buffer pH 4.5 was then injected. Residual NHS-esters were deactivated with 1 M ethanolamine (GE Healthcare). Control flow cells were activated and deactivated in the same manner but without protein ligand. Analytes were ManLAM, LM and PIMs purified from Mtb H37Rv (BEI), PI-LAM purified from *M. smegmatis* (BEI), and mannan from *Saccharomyces cerevisiae* (Sigma). The acylation state of PIMs was assessed by thin layer chromatography and mass spectrometry, revealing that the glycolipids were tri-acylated and tetraacylated (other acylation states were not significantly detected). Analytes were diluted in HBSN running buffer and injected at increasing concentrations (0.312 µM to 2.5 µM) at a flow rate of 30 µl/min. At the end of the injection, complexes were allowed to dissociate for 10 min. Chip surfaces were regenerated by 2–3 injections of 20 ml 50–90% ethylene glycol. Injections were separated by an equilibration delay of 30 min with HBSN at a flow rate of 5 ml/min. The final amount of bound analyte, expressed in resonance units (RU), was calculated by subtracting the RU of the control flow cell from the RU of the ligand-conjugated cell. Sensograms were analyzed using BIA evaluation 3.1 software (GE Healthcare). To assess binding competition by SPR, a first injection of LM (2.5 µM for 3 min) was followed by a buffer wash (∼10 min, reflecting the time necessary for the machine to switch to another injection) and then a second injection of mannan from *Saccharomyces cerevisiae* (2.5 µM for 3 min). This injection order was chosen due to the fast dissociation rate for mannan. Overlaid sensograms of the serial injections (with the signal at the start of each injection normalized) reveal the effect of prior LM-LprG binding on mannan-LprG interactions.

### Solid phase competitive binding assay

To assess the ability of mannan to compete with LAM for binding to LprG, solid phase binding assays were performed as described [34] in 96-well plates (Corning Incorporation, NY, USA) coated with ManLAM. ManLAM was added (100 ng in 50 µl of 50% ethanol per well), and plates were air-dried for 1 h at 37°C. Subsequent steps were at room temperature. Plates were washed in wash buffer (10 mM Tris-HCl, pH 7.4, 140 mM NaCl, 1 mM CaCl_2_, 0.005% Tween 20) to remove unbound ManLAM, blocked with 5% milk in wash buffer for 2 h, washed twice in wash buffer, and incubated overnight with 100 µl of NA-LprG (1 µM) that had been pre-incubated for 30 min with or without mannan (10–500 µM) in wash buffer minus Tween. The plates were washed extensively, incubated with monoclonal anti-LprG antibody (clone α-Rv1411c, NR13806, BEI) for 2 h, washed three times, incubated for 1 h with goat anti-mouse IgG conjugated to horseradish peroxidase (Cell Signaling Technology, Danvers, MA), washed three times and incubated with o-Phenylenediamine dihydrochloride (Sigma) in 0.05 M phosphate-citrate buffer, pH 5.0 (50 µl/well) in the dark for 30 min. To stop the color development reaction, 50 µl of 2N H_2_SO_4_ was added. OD_450_ was determined on a Bio-Rad plate reader.

### Flow cytometry

Mtb cultures (Mtb strains H37Rv, H37Rv Δ*lprG*, H37Rv Δ*lprG*::*lprG-Rv1410c*, H37Ra and H37Ra Δ*lprG*) were seeded to OD_600_ of 0.05 and harvested after one week. Bacteria were declumped by 10 passages through a 23-gauge needle, incubated in blocking buffer (1% heat inactivated normal rabbit serum, 0.01% Tween 80, PBS) at room temperature for 1 h, and washed twice with FACS buffer (1% BSA, 0.01% Tween 80, PBS). Bacteria were incubated for 1 h with 500 µl of FACS buffer containing rabbit anti-ManLAM antiserum (NR-13821, BEI, Manassas, VA) or control normal rabbit serum (Life Technologies, Grand Island, NY), washed twice with FACS buffer, stained for 1 h with Alexa Fluor 647-anti-rabbit IgG (Life Technologies, Grand Island, NY), washed twice with FACS buffer, fixed with 2% paraformaldehyde for 1 h at room temperature, and suspended in PBS containing 0.01% Tween 80. Anti-LAM labeling of bacteria was assessed with an LSR II flow cytometer (BD Bioscience, San Jose, CA). Data were analyzed using FlowJo software version X (TreeStar, Ashland, OR).

### Infection of macrophages and analysis of phagosome-lysosome fusion

Female 8–12 week old C57BL/6J mice were obtained from the Jackson Laboratory (Bar Harbor, ME) and housed under specific pathogen-free conditions. Bone marrow-derived macrophages were cultured as described previously [6]. Briefly, bone marrow was flushed from isolated bones in DMEM, and cell suspensions were homogenized and filtered through a 70 µm screen. Bone marrow cells were pelleted and subjected to red blood cell lysis in ACK lysing buffer (Lonza, Walkersville, MD), pelleted again, and cultured in D10F consisting of DMEM (HyClone, Logan, UT) supplemented with 10% heat-inactivated fetal bovine serum (Gibco, Carlsbad, CA), 50 µM 2-mercaptoethanol (Bio-Rad, Hercules, CA), 1 mM sodium pyruvate (HyClone), 10 mM HEPES (HyClone), 100 units/ml penicillin, 100 µg/ml streptomycin (HyClone) and 25% LADMAC cell conditioned medium as a source of M-CSF. Cultures were incubated at 37°C in a humidified, 5% CO_2_ atmosphere. Medium was changed once on day 5 of culture, and differentiated bone marrow-derived macrophages were used on day 7. Macrophages were harvested using 0.25% trypsin/0.02% EDTA (HyClone) and 250,000 cells per well were plated in D10F without penicillin/streptomycin onto autoclaved glass cover slips (Fisher Scientific, Pittsburgh, PA) for *in vitro* infections and microscopy.

Mtb strains (H37Rv, H37Rv Δ*lprG*, H37Rv Δ*lprG*::*lprG-Rv1410c*) were grown to mid-log phase (OD_600_ = 0.5 to 0.6), pelleted, suspended in PBS with 0.05% Tween 80, and declumped by vigorous vortexing. Remaining clumps were removed by centrifugation for 15 min at 100 *g*. Some aliquots of Mtb were heat-killed by incubation at 80°C for 20 min. For fluorescent labeling of bacteria, FITC (Sigma) was added from a stock solution in DMSO to a concentration of 0.2 mg/ml FITC in 1% DMSO for 30 min at 37°C with gentle rotation. Bacteria were washed three times in PBS with 0.05% Tween 80 and suspended in D10F without antibiotics. Bone marrow-derived macrophages (grown on coverslips) were incubated for 30 min with 100 nM LysoTracker Red (Molecular Probes, Carlsbad, CA) in D10F, and FITC-labeled bacteria were added to a multiplicity of infection (MOI) of 10 for 30 min at 37°C. Macrophages were washed and incubated in D10F containing 100 nM LysoTracker Red for 30 min, washed with PBS, and fixed for 1 h in 2% paraformaldehyde. Coverslips were washed three times in PBS and inverted onto ProLong Gold mounting media with DAPI (Molecular Probes). Slides were cured overnight and imaged using a Deltavision RT epifluorescence microscope. Image files were deconvolved using the Softworx software package (Applied Precision, Issaquah, WA). Co-localization was determined by identifying intracellular bacteria (FITC-labeled organisms within DAPI-labeled cells) with overlapping lysosomal marker (LysoTracker Red). Colocalization data were obtained by counting at least six independent fields from each sample, and the results are representative of two independent experiments. Control samples consisting of uninfected/LysoTracker Red-treated or infected/LysoTracker Red-untreated bone marrow-derived macrophages were analyzed to set the lower threshold for FITC and LysoTracker Red positivity, and all infected and LysoTracker-labeled macrophages were analyzed with identical intensity cutoffs.

### Statistical analysis

Statistical analyses were performed using GraphPad Prism 5.01 software (La Jolla, CA). Fisher's exact test or Students two-tailed *t*-test was used to analyze the statistical significance of differences between groups.

## Supporting Information

Figure S1 **Total LAM expression is unaffected by deletion of** ***lprG*** **.** Mtb H37Ra and H37Ra Δ*lprG* were harvested from one-week cultures. Whole cell lysates were prepared by sonication in lysis buffer and analyzed by SDS-PAGE. Mtb lipoglycan components and control proteins were detected by Western blot with (**A**) anti-LprG antibody (to confirm knockout of LprG in H37Ra Δ*lprG*). (**B**) Monoclonal anti-LAM antibody (CS-35) or (**C**) rabbit polyclonal anti-Mtb antibody. (**D**) Lipoglycans were detected by carbohydrate staining. (**E**) Western blot with anti-DnaK was used as a loading control. Blots are from one experiment and representative of at least three independent experiments.
(PDF)
