# Correction: *Mycobacterium tuberculosis* Lipoprotein LprG Binds Lipoarabinomannan and Determines Its Cell Envelope Localization to Control Phagolysosomal Fusion

**DOI:** 10.1371/journal.ppat.1004596

**Published:** 2014-12-12

**Authors:** The PLOS Pathogens Staff

The affiliation for the ninth author is incorrect. Mary Jackson is not affiliated with #5 but with #2 Mycobacteria Research Laboratories, Department of Microbiology, Immunology and Pathology, Colorado State University, Fort Collins, Colorado, United States of America.
