# Supplementary figures and images for: Mycobacterium tuberculosis Lipoprotein LprG Binds Lipoarabinomannan and Determines Its Cell Envelope Localization to Control Phagolysosomal Fusion
Source: PLoS Pathog. 2014 Oct 30;10(10):e1004471. doi: 10.1371/journal.ppat.1004471 (PMC4214796; doi:10.1371/journal.ppat.1004471)

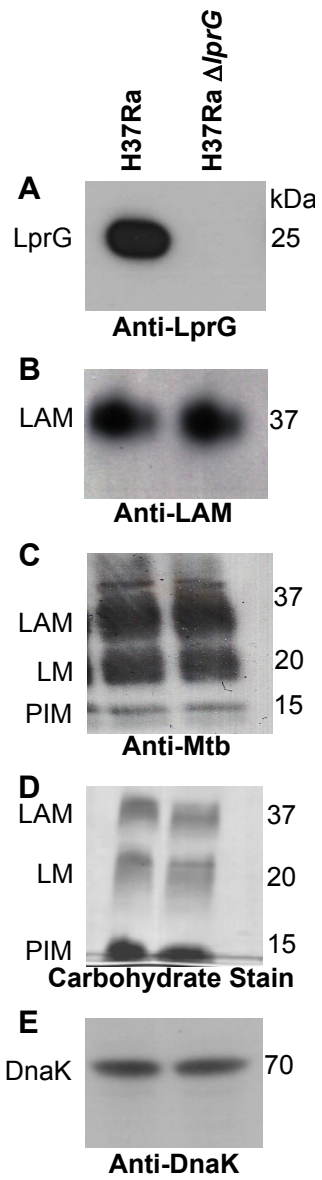

Supplement: Figure S1 — Total LAM expression is unaffected by deletion of lprG . Mtb H37Ra and H37Ra ΔlprG were harvested from one-week cultures. Whole cell lysates were prepared by sonication in lysis buffer and analyzed by SDS-PAGE. Mtb lipoglycan components and control proteins were detected by Western blot with (A) anti-LprG antibody (to confirm knockout of LprG in H37Ra ΔlprG). (B) Monoclonal anti-LAM antibody (CS-35) or (C) rabbit polyclonal anti-Mtb antibody. (D) Lipoglycans were detected by carbohydrate staining. (E) Western blot with anti-DnaK was used as a loading control. Blots are from one experiment and representative of at least three independent experiments. (PDF) [file ppat.1004471.s001.pdf]
